# Supplementary material for: Blockade of glucagon signaling prevents or reverses diabetes onset only if residual β-cells persist
Source: eLife. 2016 Apr 19;5:e13828. doi: 10.7554/eLife.13828 (PMC4871705; doi:10.7554/eLife.13828)
Supplement: Supplementary file 1. — DOI: http://dx.doi.org/10.7554/eLife.13828.019 [file elife-13828-supp1.zip › Supplementary_file_1.pdf]

Supplementary Table 1

Gene	Forward Primer (5' → 3')	Reverse Primer (5' → 3')
Actb	GGCTGTATTCCCCTCCATCG	CCAGTTGGTAACAATGCCATGT
Fbxo32	AAGGAGCGCCATGGATACTG	AGCTCCAACAGCCTTACTACG
FoxO1	GAGAAGAGGCTCACCCTGTC	ACAGATTGTGGCGAATTGAA
Gadd45a	AGACCGAAAGGATGGACACG	GGGTCTACGTTGAGCAGCTT
Gapdh	TCCATGACAACTTTGGCATTG	CAGTCTTCTGGGTGGCAGTGA
Gck	TAGCGGGGGTCATAAATCGC	GCTCCTTGAAGCTCGGGTG
Gusb	GGCGATGGACCCAAGATACC	CCATTCACCCACACAACTGC
IRS2	ATCGACTTCCTGTCCCATCA	GGCTGGTAGCGCTTCACT
Nono	TCCCTGATGCGAGAGAACAAG	GCCCATAGCACCTCCCATAG
Pepck	GATGACATTGCCTGGATGAA	CGTTTTCTGGGTTGATAGCC
p21	GCAGACCAGCCTGACAGATTT	CTGACCCACAGCAGAAGAGG
p27	CAGACGTAAACAGCTCCGAATTA	TTCAATGGAGTCAGCGATATGT
Trim63	AACTTGTGGAGACCGCCATC	CAGCCCTTGGAGGCTTCTAC
4e-bp1	CCAGCAGCCCGGAAGATAAG	GCCTTGGGGGACATAGAAGC

Supplementary Table 1: Primer sequences used for RT-qPCR.
